# Telecare motivational interviewing for diabetes patient education and support: a randomised controlled trial based in primary care comparing nurse and peer supporter delivery

**DOI:** 10.1186/1745-6215-8-18

**Published:** 2007-06-28

**Authors:** Jeremy Dale, Isabela Caramlau, Andrea Docherty, Jackie Sturt, Hilary Hearnshaw

**Affiliations:** 1 Warwick Medical School, University of Warwick, Coventry, UK; 2 Redditch and Bromsgrove PCT, Redditch, UK

## Abstract

**Background:**

There is increasing interest in developing peer-led and 'expert patient'-type interventions, particularly to meet the support and informational needs of those with long term conditions, leading to improved clinical outcomes, and pressure relief on mainstream health services. There is also increasing interest in telephone support, due to its greater accessibility and potential availability than face to face provided support. The evidence base for peer telephone interventions is relatively weak, although such services are widely available as support lines provided by user groups and other charitable services.

**Methods/Design:**

In a 3-arm RCT, participants are allocated to either an intervention group with Telecare service provided by a Diabetes Specialist Nurse (DSN), an intervention group with service provided by a peer supporter (also living with diabetes), or a control group receiving routine care only. All supporters underwent a 2-day training in motivational interviewing, empowerment and active listening skills to provide telephone support over a period of up to 6 months to adults with poorly controlled type 2 diabetes who had been recommended a change in diabetes management (i.e. medication and/or lifestyle changes) by their general practitioner (GP). The primary outcome is self-efficacy; secondary outcomes include HbA1c, total and HDL cholesterol, blood pressure, body mass index, and adherence to treatment. 375 participants (125 in each arm) were sought from GP practices across West Midlands, to detect a difference in self-efficacy scores with an effect size of 0.35, 80% power, and 5% significance level. Adults living with type 2 diabetes, with an HbA1c > 8% and not taking insulin were initially eligible. A protocol change 10 months into the recruitment resulted in a change of eligibility by reducing HbA1c to > 7.4%. Several qualitative studies are being conducted alongside the main RCT to describe patient, telecare supporter and practice nurse experience of the trial.

**Discussion and implications of the research:**

With its focus on self-management and telephone peer support, the intervention being trialled has the potential to support improved self-efficacy and patient experience, improved clinical outcomes and a reduction in diabetes-related complications.

**Trial Registration:**

Current Controlled Trials, ISRCTN63151946

## Background

Numerous studies have reported the relationship between self-management practice for long term conditions, patient behavioural change and subsequent improvement in physical and psychological well being and reduction in health service resource use [1,2]. A review of 145 papers on self management revealed a growing body of evidence demonstrating that compared with standard care, self management approaches improve knowledge, performance of self management behaviours, self efficacy and health status including reduction in blood glucose levels, calorific intake, weight and lowering serum cholesterol [3].

About 2% of the UK population are diagnosed as having diabetes, and the number is predicted to double to over 3 million by 2010 [4]. Diabetes is expensive, accounting for 8–9% of total health service costs in the UK. Much of this relates to the costs of treating the complications of diabetes, such as coronary heart disease and renal failure. Adherence in diabetes has been reported as difficult and complex [5], few patients taking their prescribed regimen entirely as intended [6]. Effective diabetes self-management training interventions have been developed, but the challenge is how to deliver these in a way that is practical, consistent, cost-effective, and acceptable to patients and health care professionals in the 'real world' [7-9]. Adherence can be increased by enabling patients to improve and reinforce understanding, combined with recognition, identification and resolution of obstacles to therapeutic advice [9]. A productive time to intervene to promote adherence is when a new therapy is introduced [10].

The role of the patient in diabetes self-management has expanded beyond responsibility for personal care to incorporate the role of educator. This latter role acknowledges the value of diabetes patient knowledge and experience in the provision of effective education and support. In the UK, in recent years the importance of developing this role has been promoted by health policy. As indicated within the Expert Patient Report [11] research findings 'display the ability of patients to successfully guide other patients in becoming 'expert' in their own care and highlight the value of patient knowledge as an educational and communicative tool'. In line with these observations and combined with the growing awareness of the benefits of lay involvement, the telephone support system of care being tested in this trial incorporated both peer and nurse support.

A potential method of delivering self-management programmes, whether lay or health professional-led, is via telephone contact. A recent Cochrane review has shown that telecommunication for patient care has been found to be acceptable to patients [12]. While telephone contact with patients is a common feature of diabetes specialist nurse practice in the UK, the effectiveness of this is unknown [13], as is the value of telephone support in diabetes care provided by peer supporters. More frequent contact with the healthcare provider may positively affect a patient's adherence to self-management activities [13], enhance autonomy and confidence in managing diabetes [14], and help the healthcare provider and the patient to revise and adjust diabetes management goals [15,16].

In summary, the behavioural change witnessed in patients as a result of improved and supported self-management practice has been associated with a range of physical and psychological benefits. When triangulated with the acceptability and cost effectiveness of telecare and the increasing awareness of the lay advisor role in the successful provision of support, the literature identifies a potentially efficacious, cost effective and patient centred method of enhancing self-management.

### Intervention development and trial design

In line with the MRC framework for development and evaluation of RCTs for complex interventions to improve health [17], the current study proposal has incorporated three key stages of research development. A theoretical basis was established from relevant literature, feasibility work was conducted to develop an understanding of the intervention and its potential outcomes and finally an exploratory trial is being undertaken to determine the requirements of the design and the success of the research strategy.

The feasibility study explored the acceptability and potential benefits of the proposed intervention via qualitative and quantitative research and consultation with expert health professionals [18]. A questionnaire was distributed to 107 GP practices within the Warwick Diabetes Care Research Framework. One hundred percent of responders (N = 82) considered the diabetes telephone care service to be 'highly' or 'very highly' important to the NHS, feasible and of potential benefit to patients. Qualitative interviews were conducted with 7 primary care and secondary care diabetes health professionals. The provision of telephone support to patients experiencing a change in treatment within primary care was considered extremely beneficial and currently, due to inadequate health service resources, a goal which was not being achieved well in either secondary or primary care. Interviewees reported that although telephone support is often provided from secondary care by diabetes specialist nurses (DSN) when patients are first changed to insulin, the short-term nature of this support meant that it had limited effectiveness in assisting patients with the long-term physical and psychological difficulties of treatment change. Additionally, this support is generally not provided to patients experiencing a change from diet to tablets or alteration in type of tablet. Interviewees felt this to be an area requiring greater attention in particular relation to patient difficulty in establishing and maintaining a successful medication regime within their day to day routine.

A needs assessment was also undertaken of self-management of people with a recent diagnosis of diabetes or change in diabetes therapy [19]. The study comprised 6 focus groups conducted with 23 participants recruited within the West Midlands through adverts in local newspapers and leaflets placed in GP clinics. The results clearly identified the perceived potential value of peer support within diabetes patient care. Peer support, facilitated from within the health care system, was felt to represent a valuable untapped resource for furthering and enabling self-management education and support. In addition, expert opinion was sought through the Warwick Diabetes Care Advisory Board, a group of nationally recognised experts of various disciplines related to diabetes care, chaired by Professor Harry Keen. This strongly endorsed the use of peer supporters to advise patients, in light of their knowledge of the current inadequacies in care provision, in particular relation to the ever increasing pressure being placed upon DSNs within secondary care.

Subsequently, a phase I exploratory trial was conducted to test the key components of the telecare intervention including training, recruitment, and practitioner role. The trial consisted of two fully trained peer supporters providing telephone service (shortened version: 2 calls over a 10 day period) to diabetes patients from a single practice experiencing a change in medication (tablet regime). The peer supporters participated in a comprehensive motivational interviewing training programme. The trial was conducted for 1 month and provided telecare support to 7 patients. Telephone record sheets were maintained throughout to ensure consistency of support. Evaluation telephone call interviews revealed that patients felt 'very comfortable' discussing their diabetes care with a peer supporter, were 'very satisfied' with the support received and were happy to receive this support in the future. Reported advantages included speaking to a non health professional who understood diabetes, having someone to listen and the provision of support during a period of uncertainty regarding medication change. Positive outcomes of the exploratory trial were also reported by participating health care professionals and the peer supporters who believed the success of the trial supported the importance of the main study.

### Aims of the Trial

The hypotheses to be tested in this study are that telecare motivational interviewing will be acceptable to patients and primary care health professionals; will be associated with improved social and psychological status; healthier lifestyle choices; and enhanced satisfaction with care.

We propose to:

1. Determine the impact on quality of care, behavioural change and patient responsiveness of a centralised, expert telecare (nurse and peer-led motivational support by telephone) intervention.

2. Compare the impact of nurse and peer supporter telecare provision against routine care.

3. Obtain clinical data as a means to identifying patterns and trends to be used in conjunction with behavioural change outcomes, in order to determine the value of undertaking a full scale RCT of the cost and clinical effectiveness of the intervention.

## Methods/Design

This study is an RCT of three arms:

i. Telecare service provided by diabetes specialist nurses

ii. Telecare service provided by peer supporters

iii. Control group receiving routine care only

Randomisation to control or intervention will be carried out at the patient level. Within the intervention group, patients will be randomly allocated to either a nurse or peer telephone supporter. To limit bias arising from patients' perception of their randomisation, all patients will receive at least one phone call. This should also ensure that the referring healthcare practitioners are as blinded as realistically possible. Patients will not be informed about whether they are in the intervention or control groups. The name, address, telephone number, access times, current treatment, details of any recent change in therapy (medication, diet, monitoring, and physical activity programme) related to diabetes, together with any provisos that were made, will be sent to the trial centre by the referring GP/nurse.

### Proposed Telecare Service

DSNs and peer supporters experienced in diabetes self management will systematically provide telephone support and advice to patients referred from primary care after a change in the patient's diabetes management (medication and/or lifestyle changes) recommended by their GP. It will be offered as an addition to routine care. The supporters will work to a protocol that includes a detailed enquiry into adherence to prescribed therapy, identification of problems, and brief negotiation in which realistic manageable goals for lifestyle change and overcoming barriers are agreed. The overarching aim of the telecare support is to reinforce the goals already established between health professional and patient.

The supporters will be recruited using a specification that sets out essential and desirable attributes established from stakeholder consultation and user group involvement. They will be trained using a 2-day programme developed by Warwick Diabetes Care. The programme is based on the theoretical background to successful behaviour change as described by Anderson and Funnell [20] and Rollnick et al [21] and practical counselling skills to enhance people's own decision making [22]. The programme includes training in empowerment, motivational interviewing, active listening skills, a brief psychological intervention that has been shown to be an effective strategy to increase readiness to change, assessing readiness to change, and the negotiation of agreed changes. Supporters will subsequently obtain an understanding of the factors that affect health behaviour, methods to assist others in altering these behaviours, and mastering the application of these techniques via telephone. Peer supporters, to further enhance their experience of patient support provision will attend a series of structured role-play sessions.

For patients in an intervention arm of the trial, the first telecare call will be made 3–5 days after referral from the patient's GP. The telecare supporter will tailor (and document) the frequency of subsequent calls to each patient's needs. The 'standard package' will be offering subsequent contact during the periods of days 7–10, 14–18, 28–35, 56–70, 120–150 but some patients will require more frequent contact while others may want less. Before completion of each call, the telecare supporter will negotiate the time of the next call. Supporters will routinely provide feedback on all calls to the research team via a standard form. However, if the telecare supporter feels more immediate contact is required, they will follow a formal feedback process to the GP; for peer supporters this will be a two stage process of referral to nurse and then, where appropriate GP. The reasons for such contact will be documented. Discussion with diabetes specialist nurses who had experience of telephone contact with patients suggested that the mean follow-up call length will be 5–10 minutes. Hence, on average each patient within the intervention groups will receive a total telecare intervention of about 45 minutes duration.

Patients in the control group will receive a single call from a researcher lasting approximately 10 minutes. This call will inform the patient that s/he is allocated to the routine care group, and that is the only phonecall s/he receives. The patient will be encouraged to follow the medical advice given to her/him by the GP/practice nurse.

#### Setting

The study is being undertaken within general practices within the West Midlands South Strategic Health Authority. This is a socio-economically and geographically diverse region in central England in which to test uptake and effectiveness of the intervention and trial methodology. In all, 40 practices have agreed to participate. Training was given to a practice nurse and general practitioner from each practice on how to manage patient recruitment, inclusion and exclusion criteria, consenting, maintaining subjects in the trial, collecting data etc.

### Setting up the telecare intervention

Twelve DSNs and nine peer supporters have been recruited using the DSN directory, Warwick Diabetes Care User Group and an email support group hosted by Trefoil Solutions to work as sessional telecare supporters for the study. In addition to interest in the study, they need to be willing to work flexible hours and be able to attend the training programme outlined above. The telecare supporters will receive monthly email or telephone supervision from their trainer to monitor workload, feasibility of the intervention, adequacy of training, and discuss specific patients presenting difficulties for the telecare supporter. A proportion of the telecare interactions will be tape-recorded. The tapes will be used to validate the data recorded during telecare interactions by the supporter, as a means to enhancing quality improvement of the intervention and for training purposes. Consent will be sought both before and after each taped interaction.

A trial centre will be established which supporters and patients can access throughout the duration of the study. Referral processes will be in place to respond to patient concerns. All patient record files will be kept under lock and key at the telecare centre and accessible only to the telecare service providers and the lead researchers. Wherever possible, anonymised patient data will be used. Confidentiality will be maintained and all provisions of the Data Protection Act will be followed.

### Inclusion/Exclusion Criteria

The inclusion/exclusion criteria were initially set as:

#### Eligible patients

Patients who have had a recent HbA1c greater than 8.0% and been advised of the need to improve their glycaemic control, with or without a change in prescribed tablet based therapy.

#### Exclusions

Patients on insulin, lacking a telephone, and those with severe accompanying disorders (e.g. mentally ill; severe learning difficulties; severe hearing difficulties) or disorders judged by the GP as likely to interfere with outcome interpretation. Exclusions will *not *be made on the basis of anticipated patient co-operation, except where there are objective reasons for so doing.

10 months into the trial, the clinical threshold for inclusion was lowered in response to requests from GPs to allow patients with baseline HbA1c > 7.4% (as opposed to the previous criteria of HbA1c > 8%) to become eligible for telecare. This modification to the protocol is in line with the current NHS targets (new GMS contract target is 7.4%) and the manner in which practices are organising and delivering their care. The latest research evidence published in the American journal *Diabetes Care *also supports this change in eligibility criteria. In addition, the change was supported by research findings published by Young et al (2005) which reported significant improvement in HbA1c values as a result of telecare for patient with baseline HbA1c > 7% [23].

#### Recruitment of patients

At each practice site, the lead GP or practice nurse for diabetes is responsible for managing patient recruitment. During the data collection period, all adult patients with type 2 diabetes with an impending review appointment who meet the inclusion criteria will be contacted by post and invited to participate within the study. Participation and consent are subsequently discussed with the referring GP or nurse at the review appointment. Hence, the intention is that the intervention should be implemented systematically within the normal practice routine, as far as possible. Patients will be provided with written information about the study, and invited to give written consent for inclusion.

### Experiments Proposed Including End Points

The hypotheses to be tested are that the telecare intervention compared with routine care alone will improve adherence to the change in medication targeted; improve clinical and psychological status; encourage healthier lifestyle choices and enhance satisfaction with care. In addition, that it will be acceptable to patients and primary care health professionals.

#### Baseline Data

In addition to the questionnaires outlined below, for patients who consent to be randomised, sex, age, ethnicity, diabetes history, most recent clinical parameters (HbA1c, BP, BMI, total plasma cholesterol, HDL: total cholesterol ratio, and plasma triglycerides), prescribed medication, smoking status, phone number, access hours, and any provisos requested by the patient will be recorded on a pro forma at recruitment by practice staff. Patients who do not wish to participate in the study will be asked for consent to include their reasons for not doing so, and their sex, age, ethnicity, most recent clinical parameters and smoking status will be recorded. This will allow for comparison between the characteristics of participants and non-participants.

#### Outcome Data

All outcome data from patients in the intervention and control groups are being collected at site level by a practice nurse or GP, recorded on templates developed for the purpose, and forwarded by post to the trial office. Patients in the intervention groups will be asked to complete a 4 page questionnaire after 3 and 6 months, while patients in the control group will complete the questionnaire only at 6 months. The questionnaire incorporates The Problem Areas in Diabetes Scale (PAID) [24], the Diabetes Management Self-Efficacy Scale (DMSES) [25], the diabetes self-care activities measure [26], the Perceived Therapeutic Efficacy Scale (scale under development) [27], and an acceptability of service provision measure. The questionnaire will be completed at the surgery and posted in a reply paid envelope.

#### Clinical Data

Clinical data at baseline will be collected prior to patient randomisation. Blood specimens for HbA1c assessment, total and HDL cholesterol are being taken according to local protocols either by practice staff or the local phlebotomy service. They are being analysed by the local hospital based laboratory services routinely used by the practices, blinded to patient group allocation. We will ensure that if changes in standardisation occur during data collection that, at each site, all data are reported according to the same standard. Practices will arrange a 3 and 6 month review appointment with patients in the intervention group and a 6 month review for those in the control group at which point clinical follow up data will be collected. A reminder of forthcoming appointments will be distributed by the trial office to each participating practice 2 weeks before the appointment is due.

#### Content of Telecare Intervention

Telecare nurses and peer supporters will record the dates, times and duration of calls made to each patient, and the number of attempts required to make contact with the patient on each occasion. The content of calls made to and received from patients (including referrals) will be downloaded from the electronic template. Patient dropouts and reasons for this will also be recorded.

#### Sample size

The sample size is based upon the primary outcome measure of self efficacy, measured using the DMSES and has been constructed to detect a difference in the primary outcomes of self efficacy perceptions and psychological adjustment. A sample of at least 300 patients (minimum 100 in each arm) has been constructed to detect a difference in self-efficacy scores with an effect size of 0.35, 80% power at a level of 5% significance level [25]. Allowing for an attrition rate of 25% from initial recruitment, we will require 125 subjects in each arm (i.e. 375 participants in total).

### Ethics approval

The study has received approval from Warwickshire LREC (reference number: RE 610).

### Trial Analysis

Data will be entered in SPSS. A random 20% of the data will be entered twice to check for discrepancies with the original data. Descriptive methods will be used to demonstrate the consistency of the three groups, describe participant characteristics, to compare the refusers with the study participants and report levels of participation and drop out. Comparison of nurse and peer supporter outcome data, experiences and feedback will be provided. Analysis of variance will be used to evaluate baseline characteristics of participants compared to non-participants, overall significance of improvement across outcome measures, and dropout versus maintainers. For each patient, change in physiological measures from start to 6 months will be calculated and analysed using t-test for matched groups for both intervention and control groups. The magnitude of change in both primary and secondary outcome measures will be estimated and the 95% confidence intervals given. *Formal Statistical Input*: A research fellow in medical statistics will provide statistical support and advice throughout the study.

### Qualitative studies

Several qualitative studies are being conducted in parallel with the main RCT:

1. Telecare supporters' experience: telephone semi-structured interviews with the supporters (diabetes specialist nurses and peer supporters) will be conducted one-year into the intervention. The interviews will aim to capture their experience of delivering the intervention, and the impact of helping others on their own physical and psychological well-being (as well as their professional practice, for nurses).

2. Patient experience: telephone semi-structured interviews will be undertaken with a purposive sample of patients allocated to each intervention arm of the trial, to include patients who have improved HbA1c from baseline to 6 months together with others who have failed to improve. The interview will explore participants' views about the service, issues around the provider (e.g. credibility, trustworthiness, helpfulness), elements of the intervention, and benefits of the service.

3. Referring healthcare professional experience: telephone semi-structured interviews will be conducted with twelve GPs and practice nurses following completion of the six-month data collection, in order to capture their views on self-management, experience of patient recruitment and participation in the research study.

The interviews will be recorded and transcribed verbatim. A thematic framework approach will be used to analyse the data [28]. The framework approach is a systematic process of analysing the data, following a clear procedure and allowing the material to be categorised according to key themes. First, in the familiarisation stage, a relevant sample of tapes will be listened to, the transcripts will be read, and notes will be made on key issues and recurrent themes. In the second stage, the notes will be used to set up a thematic framework, based on a priori issues (the interview topic guide), as well as recurring views and emergent issues raised by the participants. In the third stage, the index will be applied to all interview transcriptions, followed by the fourth stage, where data will be rearranged according to themes (process known as charting). In a final stage, data will be mapped and interpreted. The data will be analysed independently by 2 researchers. Where disagreement occurs, consensus will be reached through discussion.

## Discussion

### The Value of the Research to Public Health and Patient Care

It is anticipated that the provision of motivational telecare support will enhance the self-management of patients with type 2 diabetes leading to improved clinical and psychological outcomes and reduction in the likelihood of diabetic complications. In addition to the improvement in individual patient care, the service could provide a cost effective adjunct to routine care, alleviating the resource pressures currently experienced within primary care and secondary care practice while establishing a successful and robust method of responding to the increasing prevalence of diabetes.

The project, in light of its focus upon self-management, peer involvement and telephone support has the potential to demonstrate an approach that is relevant to realising progress towards the achievement of the care targets in relation to not only diabetes, but a range of long term conditions. The intervention offers a valuable tool in dealing with the difficulties and complications of long term conditions, and as such might be applicable in a wide range of settings.

The main trial started in autumn 2004, shortly after the introduction of the new General Medical Services contract for general practice and the implementation of the Quality and Outcomes framework (QOF) [29]. The new GMS contract substantially increased the workload for general practitioners, making participation in research less attractive, and this has had an adverse impact on patient recruitment. However, the QOF targets resulted in considerably more data on diabetes care being routinely collected by GPs for the annual QOF reports, and at the same time the QOF introduce financial incentives to bring the patients' HbA1c levels below the 7.4% threshold through the more aggressive prescription of oral hypoglycaemic agents and insulin. Together, this resulted in slow participant recruitment for the trial, due to a reduction in the number of eligible patients. In response, the clinical threshold for trial inclusion was lowered to allow patients with baseline HbA1c > 7.4% (as opposed to the previous criteria of HbA1c > 8%) to become eligible for telecare, in line with the manner in which practices are organising and delivering their care. The change in protocol mid-way through the trial will have implications on the presentation of the consort diagram and the interpretation of the study's findings.

The qualitative studies conducted in parallel with the main trial aim to capture the views of patients, supporters and health care professionals on their involvement in the study, satisfaction and benefits derived. It will enrich the interpretation of the quantitative data obtained from the main trial, by shedding light on the importance of various components of this complex intervention. Together with the quality assurance procedures, the qualitative studies will help to inform future intervention delivery research, particularly in the areas of patient and peer supporter recruitment, training delivery, the use of the theoretical framework within the intervention delivery, and the experience and potential benefits of telephone support for both patients and the supporters.

## Competing interests

The author(s) declare that they have no competing interests.

## Authors' contributions

JD, AD, JS and HH participated in the conception and design of the study. IC and JD were involved in drafting the manuscript, as well as the ethics committee and R&D applications, the acquisition of data and the coordination of the trial. JD, JS and HH participated in the critical revision of the manuscript and the trial management. All authors read and approved the final version of the manuscript.
